# Correction: Salt-inducible kinase 3 regulates the mammalian circadian clock by destabilizing PER2 protein

**DOI:** 10.7554/eLife.66683

**Published:** 2021-01-25

**Authors:** Naoto Hayasaka, Arisa Hirano, Yuka Miyoshi, Isao T Tokuda, Hikari Yoshitane, Junichiro Matsuda, Yoshitaka Fukada

Hayasaka N, Hirano A, Miyoshi Y, Tokuda IT, Yoshitane H, Matsuda J, Fukada Y. 2017. Salt-inducible kinase 3 regulates the mammalian circadian clock by destabilizing PER2 protein. *eLife* **6**:e24779. doi: 10.7554/eLife.24779.

Published 11, December 2017

We have determined that the data in Figure 1b in the original article inadvertently overlapped with a figure in a previous paper. As we described in the Acknowledgements in the original paper, the data (Figure 1b) had originally been provided by former collaborators. We did not realize that the data had already been included in another paper antecedently published by the former collaborator. We therefore removed Figure 1b from the original paper and corrected Figure 1 is shown here. Note that this correction does not affect any results or conclusion of the original paper.

**Figure 1:**
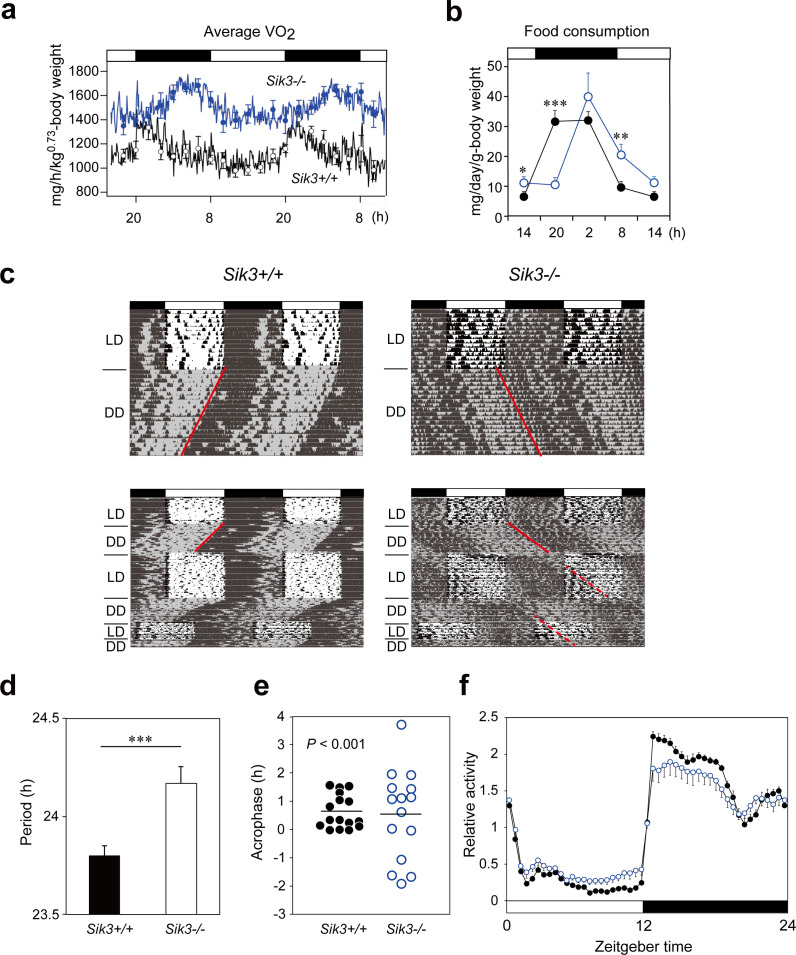


The originally published Figure 1 is also shown here for reference:

**Figure 2:**
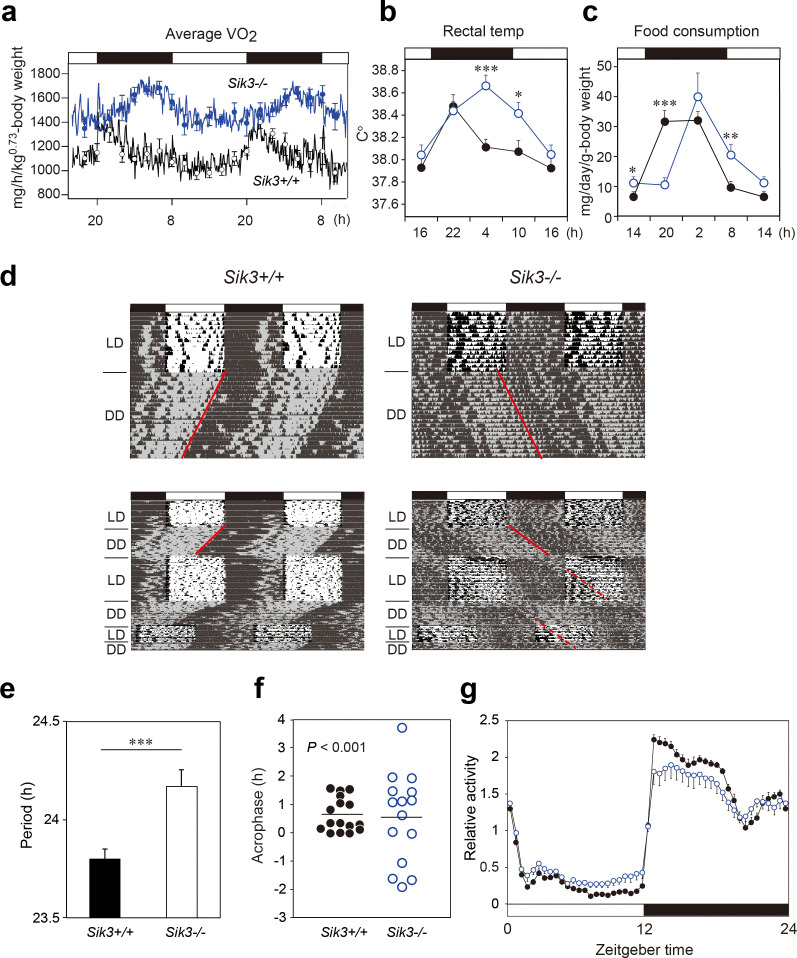


We have also corrected the Figure 1 legend according to the deletion of Figure 1b.

Corrected sentences from Figure 1 legend:

Figure 1. *Sik3^-/-^* mice exhibit aberrant circadian rhythms in physiology and behavior.

a. Oxygen consumption rhythm was measured in *Sik3*^-/-^ mice and *Sik3^+/+^* controls for two days (*n* = 3 per group), and mean values are shown. The peak in the *Sik3*^-/-^ mice (blue line) was phase-delayed by approximately 6 h compared with that of *Sik3^+/+^* mice (black line). b. Food consumption rhythm also exhibited delayed phase (*n* = 3 per each). Note that continuous food intake was observed in the *Sik3*^-/-^ mice during the resting phase (daytime). c. Locomotor activity rhythms in the *Sik3*^-/-^ mice and *Sik3^+/+^* controls. Upper panels demonstrate impaired entrainment to the light-dark (LD) cycles, low amplitude, variable phases, and significantly longer free-running periods in *Sik3*^-/-^ mice compared with *Sik3*^+/+^ controls (see angles of red lines). Lower panels show light re-entrainment experiments. In contrast to *Sik3*^+/+^ mice, in which behavioral rhythms entrained to LD cycles after transition from constant dark (DD) to LD, it took more than 2 weeks for *Sik3*^-/-^ mice to become entrained to LD. In addition, a portion of the activity rhythms in DD persistently free-ran for as long as 3 weeks after conversion from DD to LD in the *Sik3*^-/-^ mice (see dotted lines). d. Average free-running periods in the *Sik3*^-/-^ mice (*n* = 13) and *Sik3*^+/+^ mice (*n* = 15). e. Distribution of average acrophase in individual *Sik3*^-/-^ (*n* = 15) and *Sik3*^+/+^ (*n* = 16) mice. *p* < 0.001 for the test for equality of variance (F-test) vs. *Sik3*^+/+^. f. Averaged activity plots of *Sik3*^+/+^ mice (black line, *n* = 16, average of 16 days) and *Sik3*^-/-^ mice (blue line, *n* = 15) in LD. *p < 0.05, **p < 0.01, ***p < 0.001 vs. *Sik3*^+/+^ (Student’s *t*-test).

Original sentences from Figure 1 legend for reference:

Figure 1. *Sik3^-/-^*mice exhibit aberrant circadian rhythms in physiology and behavior.

a. Oxygen consumption rhythm was measured in Sik3-/- mice and Sik3+/+ controls for two days (n = 3 per group), and mean values are shown. The peak in the Sik3-/- mice (blue line) was phase-delayed by approximately 6 h compared with that of Sik3+/+ mice (black line). b. Rectal temperature rhythm in the Sik3-/- mice was phase-delayed (n = 3 each). c. Food consumption rhythm also exhibited delayed phase (n = 3 per each). Note that continuous food intake was observed in the Sik3-/- mice during the resting phase (daytime). d. Locomotor activity rhythms in the Sik3-/- mice and Sik3+/+ controls. Upper panels demonstrate impaired entrainment to the light-dark (LD) cycles, low amplitude, variable phases, and significantly longer free-running periods in Sik3-/- mice compared with Sik3+/+ controls (see angles of red lines). Lower panels show light re-entrainment experiments. In contrast to Sik3+/+ mice, in which behavioral rhythms entrained to LD cycles after transition from constant dark (DD) to LD, it took more than 2 weeks for Sik3-/- mice to become entrained to LD. In addition, a portion of the activity rhythms in DD persistently free-ran for as long as 3 weeks after conversion from DD to LD in the Sik3-/- mice (see dotted lines). e. Average free-running periods in the Sik3-/- mice (n = 13) and Sik3+/+ mice (n = 15). f. Distribution of average acrophase in individual Sik3-/- (n = 15) and Sik3+/+ (n = 16) mice. p < 0.001 for the test for equality of variance (F-test) vs. Sik3+/+. g. Averaged activity plots of Sik3+/+ mice (black line, n = 16, average of 16 days) and Sik3-/- mice (blue line, n = 15) in LD. *p < 0.05, **p < 0.01, ***p < 0.001 vs. Sik3+/+ (Student’s t-test).

The main text including Results, Discussion and Acknowledgements has also been corrected accordingly. Examples are shown here. These changes do not affect any conclusions.

Corrected sentence from Results:

We also found that other physiological rhythms related to food consumption were phase-delayed to similar degrees (Fig. 1b).

Original sentence from Results:

We also found that other physiological rhythms related to rectal temperature and food consumption were phase-delayed to similar degrees (Fig. 1b, c).

Corrected sentence from Acknowledgements:

The authors are grateful to Drs. Hiroshi Takemori and Minako Koura for providing *Sik3* KO mice and their data on metabolic rhythms (Fig. 1a, b),...

Original sentence from Ackowledgements:

The authors are grateful to Drs. Hiroshi Takemori and Minako Koura for providing *Sik3* KO mice and their data on metabolic rhythms (Fig. 1, a-c),...

